# Comparison of cerebral and cutaneous microvascular dysfunction with the development of type 1 diabetes

**DOI:** 10.7150/thno.33738

**Published:** 2019-08-12

**Authors:** Wei Feng, Shaojun Liu, Chao Zhang, Qing Xia, Tingting Yu, Dan Zhu

**Affiliations:** 1 Britton Chance Center for Biomedical Photonics, Wuhan National Laboratory for Optoelectronics-Huazhong University of Science and Technology, Wuhan, Hubei 430074, China; 2 MoE Key Laboratory for Biomedical Photonics, Huazhong University of Science and Technology, Wuhan, Hubei 430074, China

**Keywords:** type 1 diabetes, microvascular dysfunction, vascular response, optical clearing, optical imaging.

## Abstract

**Rationale:** Diabetes can lead to cerebral and cutaneous vascular dysfunction. However, it is still unclear how vascular function changes with the development of diabetes and what differences exist between cerebral and cutaneous vascular dysfunction. Thus, it is very important to monitor changes in cerebral and cutaneous vascular function responses *in vivo* and study their differences during diabetes development.

**Methods:** With the assistance of newly developed skull and skin optical clearing techniques, we monitored the responses of sodium nitroprusside (SNP)- and acetyl choline (ACh)-induced cerebral and cutaneous vascular blood flow and blood oxygen in diabetic mice *in vivo* during the development of type 1 diabetes (T1D) by combining laser speckle contrast imaging with hyperspectral imaging. We then compared the differences between cerebral and cutaneous vascular responses and explored the reasons for abnormal changes induced in response to different vascular beds.

**Results:** In the early stage of diabetes (T1D-1 week), there were abnormal changes in the cerebral vascular blood flow and blood oxygen responses to SNP and ACh as well as cutaneous vascular blood oxygen. The cutaneous vascular blood flow response also became abnormal from T1D-3 weeks. Additionally, the T1D-induced abnormal blood flow response was associated with changes in vascular myosin light chain phosphorylation and muscarinic acetylcholine receptor M3 levels, and the aberrant blood oxygen response was related to an increase in glycated hemoglobin levels.

**Conclusion:** These results suggest that the abnormal cutaneous vascular blood oxygen response occurred earlier than the blood flow response and therefore has the potential to serve as a good assessment indicator for revealing cerebrovascular dysfunction in the early stage of diabetes.

## Introduction

Type 1 diabetes (T1D) is characterized by deficient insulin production, which directly causes hyperglycemia 1. The abnormal secretion of insulin and elevated blood glucose levels can lead to severe vascular complications in various organs, including cardiovascular disease, retinal vasculopathy, cerebrovascular disease and peripheral vascular disease, etc. In particular, diabetes frequently induces cerebral ischemic injury, which mainly manifests as small vessel diseases and increases the risk of stroke, cognitive decline and dementia 2-4; therefore, researchers have paid a great deal of attention to diabetes-induced cerebrovascular dysfunction 5-7.

At present, many studies on the cerebrovascular dysfunction caused by diabetes have mainly focused on* ex vivo* or* in vitro* experiments based on isolated middle cerebral artery experiments or cultured vascular cells 5, 6. *In vivo* studies have usually employed magnetic resonance imaging (MRI) to measure the cerebral blood flow level of diabetic patients in the resting state 7. These studies are focused only on the comparison of diabetic and normal states, and little attention has been paid to studying the dynamic changes in cerebrovascular dysfunction that occur during the development of diabetes. Diabetes is a chronic metabolic disease 8, and it is therefore necessary to monitor changes in cerebrovascular dysfunction *in vivo* during different developmental stages of diabetes. This would be helpful for improving understanding of the influence of progressive diabetes on cerebral vascular dysfunction and developing interventional therapeutic strategies for diabetes. However, obtaining techniques that allow the *in vivo* noninvasive monitoring of cerebrovascular functional responses with high spatial and temporal resolution under pathological conditions has been a challenge.

The skin, as an accessible and generalized vascular bed, is attracting more attention. Our recent studies have shown that diabetes can cause abnormalities in cutaneous microvascular functions 9 and the motility of immune cells 10. Previous investigations indicated that the peripheral skin has substantial potential to be a sufficient research biomarker for predicting cardiovascular diseases 11-13 and diabetic retinopathy 14 as well as a prognostic marker for evaluating the efficacy of drugs on the microcirculation 15-17. Taylor S. L.* et al*. and Costa R.* et al*. found that the characteristics of diabetes-induced abnormal angiogenesis in the brain were consistent with changes in skin based on the expression of vascular endothelial growth factor 18, 19. However, it is unclear whether the skin vascular bed, with its rich vascular network, can serve as a research target for revealing diabetes-induced cerebrovascular dysfunctions. It remains very difficult to monitor changes in cutaneous microvascular function *in vivo* and analyze the time sequence of cutaneous microvascular reactivity tests with high resolution 15.

Optical imaging technologies, because they are noninvasive and effective, allow the monitoring of hemodynamic changes in cerebral and cutaneous microvessels. However, the high scattering characteristic of tissue limits the performance of optical imaging. To overcome these barriers, many imaging windows have been established in surgery to improve imaging performance, and these include various skin windows 20, 21 and skull windows 22-24. Surgery inevitably causes local and even systemic pro-inflammatory stimuli, which can lead to changes in vascular structure and function 25, 26. With the development of tissue optical clearing techniques, methods for *in vivo* optical clearing skin and skull windows have been proposed 27-29. The imaging performance of various optical imaging modalities, such as optical coherence tomography 30-32, photoacoustic microscopy 33, laser speckle contrast imaging (LSCI) 34-39, hyperspectral imaging (HSI) 28, 40, 41 and confocal microscopy 27, have substantially improved.

This work aims to evaluate cerebral and cutaneous vascular dysfunctions during diabetes development and study the relationships between them. Here, *in vivo* skull and skin optical clearing techniques permitted us to monitor sodium nitroprusside (SNP)- and acetyl choline (Ach)-induced cerebral and cutaneous microvascular responses *in vivo* (including blood flow and blood oxygen) at different stages of T1D by combining LSCI with HSI. We compared the differences in T1D-induced cerebral and cutaneous vascular dysfunctions and further explored the reasons for the observed changes in vasodilation towards different vascular beds.

## Materials and Methods

### T1D model

Adult male Balb/c mice (8 weeks old, 20±2 g) were intraperitoneally administered with 150 mg/kg alloxan (30 mg/ml) for four days at 4 hours after fasting each day. The consecutive detection of the blood glucose will be performed for validating the T1D mice model, which depends on whether the fasting blood glucose is higher than 7 mmol/L. T1D mice were divided into four groups depending on their diabetes duration, namely 1-, 2-, 3- and 4-week T1D mice used for experiments. All experimental procedures were performed according to animal experiment guidelines of the Experimental Animal Management Ordinance of Hubei Province, P. R. China, and the guidelines from the Huazhong University of Science and Technology, which have been approved by the Institutional Animal Ethics Committee of Huazhong University of Science and Technology.

### *In vivo* skin optical clearing technique

To improve optical imaging quality, we employed a recently developed *in vivo* skin optical clearing technique. Here, to monitor subcutaneous vessels with high resolution, an optical cleared dorsal skin window was established via topical treatment of the dorsal skin with an optical clearing agent, which was mixed with polyethylene glycol, thiazone and sucrose (mass fraction: 67.1%) at a volume ratio of 9:1:10 according to a previous study 27. The hair was shaved and any residual hair was removed thoroughly via treatment with a depilatory cream. Then, the skin optical clearing agent was topically applied on the dorsal region of interest for 15 min, and this quickly made the skin optical transparent and effectively improved imaging contrast. The details about our usage of *in vivo* skin optical clearing agents can are described in our previous study 27.

### *In vivo* skull optical clearing technique

In addition, an innovative optical clearing skull window was employed to noninvasively monitor cortical vessels via treatment with a skull optical clearing agent, which consisted of two solutions (solution-1 and solution-2). Solution-1 was a saturated supernatant solution consisting of 75% (vol/vol) ethanol and urea at 25 °C and was applied to the exposed skull of mice for 10 min to make the skull transparent. Solution-2 is a high-concentration sodium dodecyl benzene sulfonate solution that was prepared by mixing 0.7 M NaOH solution with dodecyl benzene sulfonic acid at a volume-mass ratio of 24:5. Solution-2 was applied after solution-1 to enhance skin clearing and was applied for 5 min. The detailed protocol describing our use of these *in vivo* skull optical clearing agents is provide in our recent study 28.

### Dual-mode system for monitoring blood flow and oxygen responses

**Figure 1:**
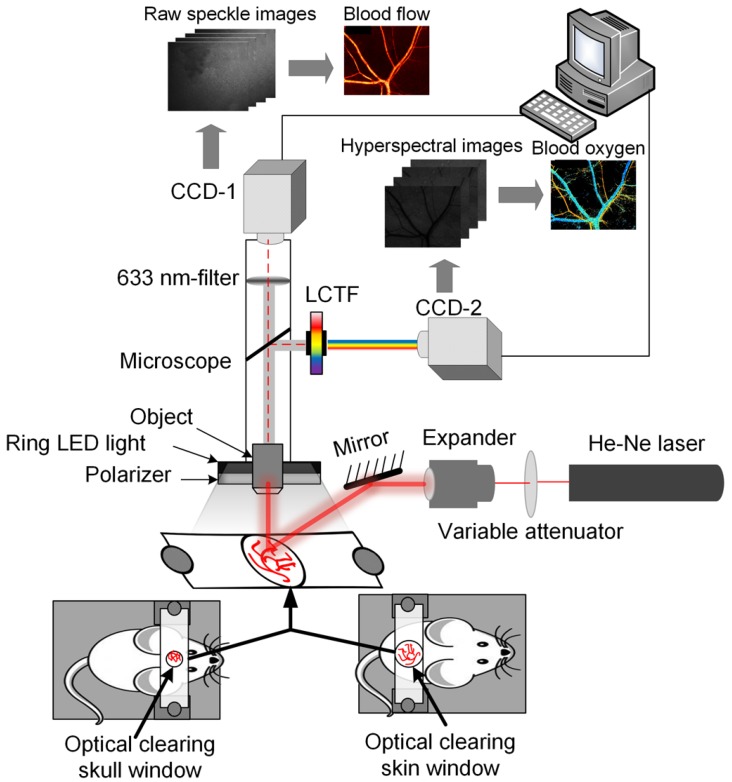
** System diagram for LSCI & HSI.** Monitoring of vascular blood flow and blood oxygen through optical clearing windows using LSCI and HSI dual-mode systems (CCD-1 was used to acquire the data used to obtain blood flow maps, and CCD-2 was employed to acquire spectral images to obtain blood oxygen maps.)

Here, the dual-mode system was developed for simultaneously monitoring blood flow and blood oxygen by combining LSCI and HSI. **Figure 1** shows a schematic of the system. The system mainly consisted of two charge-coupled device (CCD) cameras (Pixelfly USB, PCO Company, Germany), CCD-1 was used to acquire LSCI raw data, and CCD-2 was used to acquire HSI raw data. In addition, it consisted of a liquid crystal tunable filter (LCTF, CRi Varispec VIS, Perkin Elmer, USA), a stereo microscope (SZ61TR, Olympus, Japan), a ring-like LED light with a polarizer, a filter (633±5 nm) and an He-Ne laser beam (λ = 632.8 nm) expanded by a collimating lens to illuminate the areas of interest. The bandwidth of LCTF was 7 nm. A certified reflectance standard (SRS-99-020, Labsphere, USA) was used to obtain standard hyperspectral images. To obtain the absorption of hemoglobin, hyperspectral images were acquired from 500 nm to 620 nm (with a step size of 10 nm). Vascular blood oxygen saturation was calculated based on a hyperspectral dataset using a previously described algorithm 40. Strong absorption of oxy-hemoglobin and deoxyhemoglobin was observed at 540 nm; therefore, both the artery and the vein could be clearly observed in the images. At 620 nm, the absorption of deoxyhemoglobin is much higher than that of oxy-hemoglobin, and only the vein with rich deoxyhemoglobin was visible in the image. Hence, the artery and vein could be distinguished 40. Finally, the corresponding blood flow velocity was obtained using the laser speckle temporal contrast analysis method 42.

Using this dual-mode system, we monitored cerebral and cutaneous blood flow and blood oxygen in the resting state during the first 10 min at 0.5-min intervals. Then, 125 μL of SNP (0.2 mg/mL) or 125 μL of ACh (0.4 mg/mL) was injected via the vein, and we recorded images for 30 min at 0.5-min intervals to observe the dynamic changes induced by this reactive test. Different optical clearing windows were established in different mice, and cerebral and cutaneous vascular responses were monitored separately in different mice. During the experiments, the mice were placed on a heating pad to maintain the body temperature of the mice at 36.5 - 37.5 °C. To avoid the effects of various experimental factors, all experimental conditions were kept as consistent as possible.

### Evaluating the expression levels of phosphorylated myosin light chain (P-MLC) and muscarinic acetylcholine receptor M3 (M3R)

Further, to explore the reasons for changes in SNP and ACh-induced vasodilation towards different vascular beds, the related protein was extracted from fresh *ex vivo* dorsal skin and brain samples of mice, which were used for western-blot analysis of P-MLC and M3R with a rabbit anti P-MLC antibody (1:1000, Cell Signaling Technology, Inc. CST#3674), M3R antibody (1:1000, Abcam, #abcam126168), mouse anti-β-actin antibody (1:8000, Tianjin Sungene Biotech Co., #KM9001) and mouse anti-GAPDH antibody (1:10000, Tianjin Sungene Biotech Co., Ltd. #KM9002).

### Measurement of physiological indexes in animals

The mean arterial blood pressure (MABP), heart rate (HR) and blood volume of mice were measured by the CODA high throughput noninvasive blood pressure system (CODA-HT8, Kent Scientifc corporation, United States).The weight of mice was measured by the electronic balance (PPT-B2000, HZ Electronic LLC, United States). In order to measure the blood glucose and glycated hemoglobin, collection of blood samples from the mice was performed at 4 hours after fasting. Then, the fresh blood was kept in the blood collection tube with anticoagulant. The blood glucose levels were determined by the Accu-Chek Active test strips (Roche Co., Germany). The glycated hemoglobin levels were determined by the Tosoh Automated Glycohemoglobin Analyzer (TOSOH HLC-723G8, TOSOH Co., Japan).

### Insulin administration

The dosage of insulin was 0.003 U/g. The injected solution was delivered by subcutaneous injection in 2-week T1D mice twice daily (10:00 am; 17:00 pm) for one week.

### Statistical analysis

In this work, the diameter of cerebral vessels for statistical analysis is from 50 μm to 140 μm, as for cutaneous vessels, the diameter is from 50 μm to 200 μm. Significant differences between T1D and non-T1D are analyzed using Student's t test with MATLAB.

## Results

### SNP-induced cerebral and cutaneous microvascular function responses

**Figure 2:**
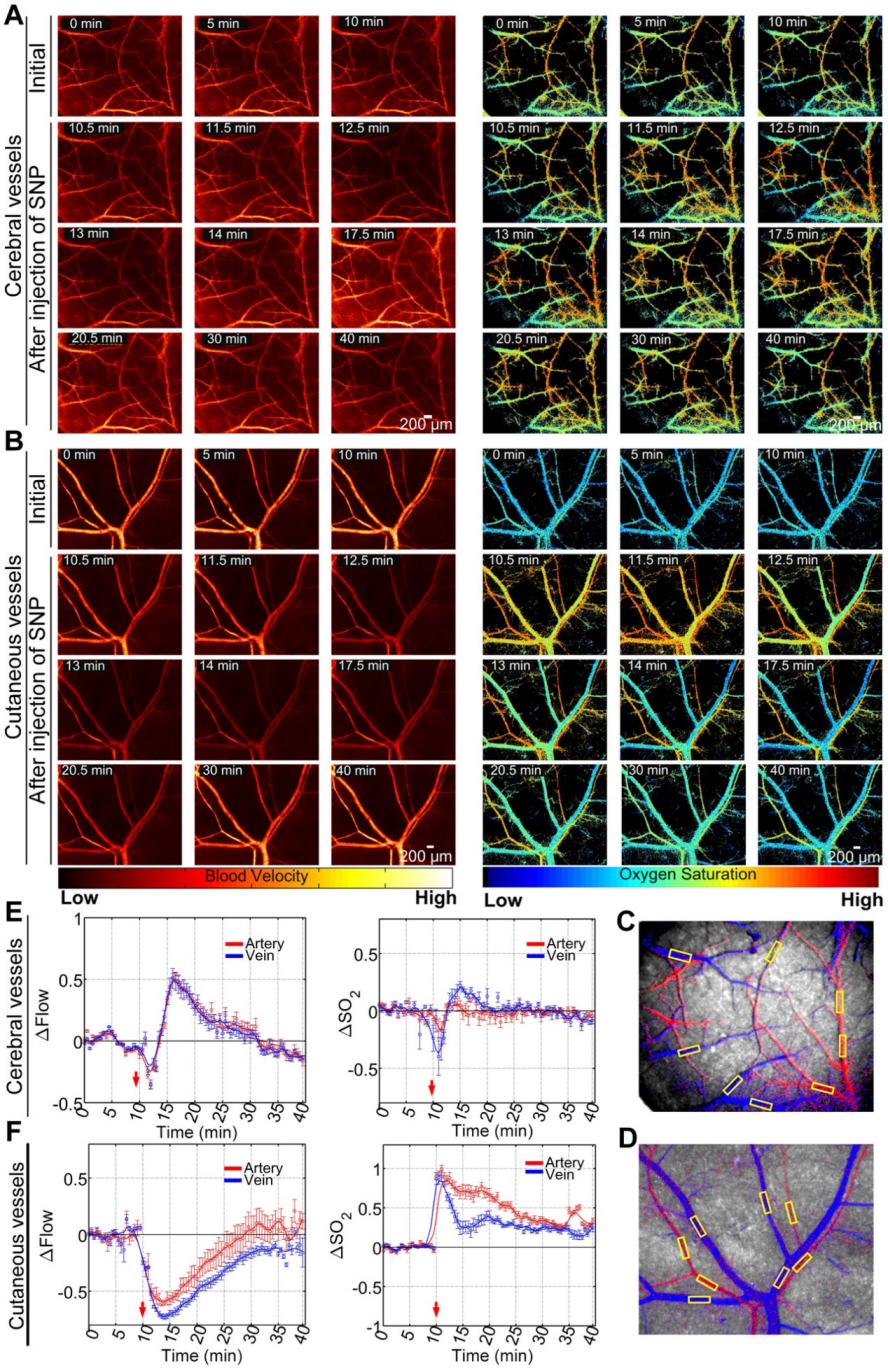
** Maps of dynamic responses of blood flow and blood oxygen saturation in non-T1D mice (1 week). (A)** Typical blood flow and blood oxygen saturation maps of cerebral vessels viewed through an optical clearing skull window before and after the injection of SNP. **(B)** Typical blood flow and oxygen saturation maps of cutaneous vessels viewed through an optical clearing skull window before and after the injection of SNP. Arteriovenous segmentation maps of cerebral **(C)** and cutaneous **(D)** microvessels (red: artery; blue: vein; yellow rectangular boxes were used for statistical analysis). Statistical analyses based on the yellow rectangular boxes containing vessel positions were performed to show the time courses of relative changes in blood flow and blood oxygen saturation in cerebral **(E)** and cutaneous **(F)** microvessels (mean ± standard error).

The optical clearing skull and skin windows allowed us to visually monitor SNP-induced cerebral and cutaneous vascular functional responses through the skull and skin, as shown in **Figure 2A-B**, respectively. Cerebral and cutaneous arteriovenous segmentation maps are presented in **Figure 2C**-**D**, respectively. Furthermore, the relative changes in arteriovenous blood flow (△Flow) and blood oxygen saturation (△SO_2_) before and after the injection of SNP were quantitatively analyzed, as shown in **Figure 2E**-**F**. In non-T1D mice (**Figure 2E**), both cortical blood flow and blood oxygen saturation were stable before injection. After SNP was injected, the △Flow quickly decreased before increasing and almost recovered to the initial level with time. Compared to △Flow, △SO_2_ showed only minor changes. Additionally, **Figure 2F** demonstrates that cutaneous microvascular △Flow gradually decreased after the injection of SNP and then gradually recovered to the initial level. Moreover, the corresponding cutaneous microvascular △SO_2_ sharply increased. As time went on, △SO_2_ gradually decreased, but it did not recover to the initial level throughout the 40-min observation period.

### Cerebral arteriovenous functional response during the development of T1D

**Figure 3:**
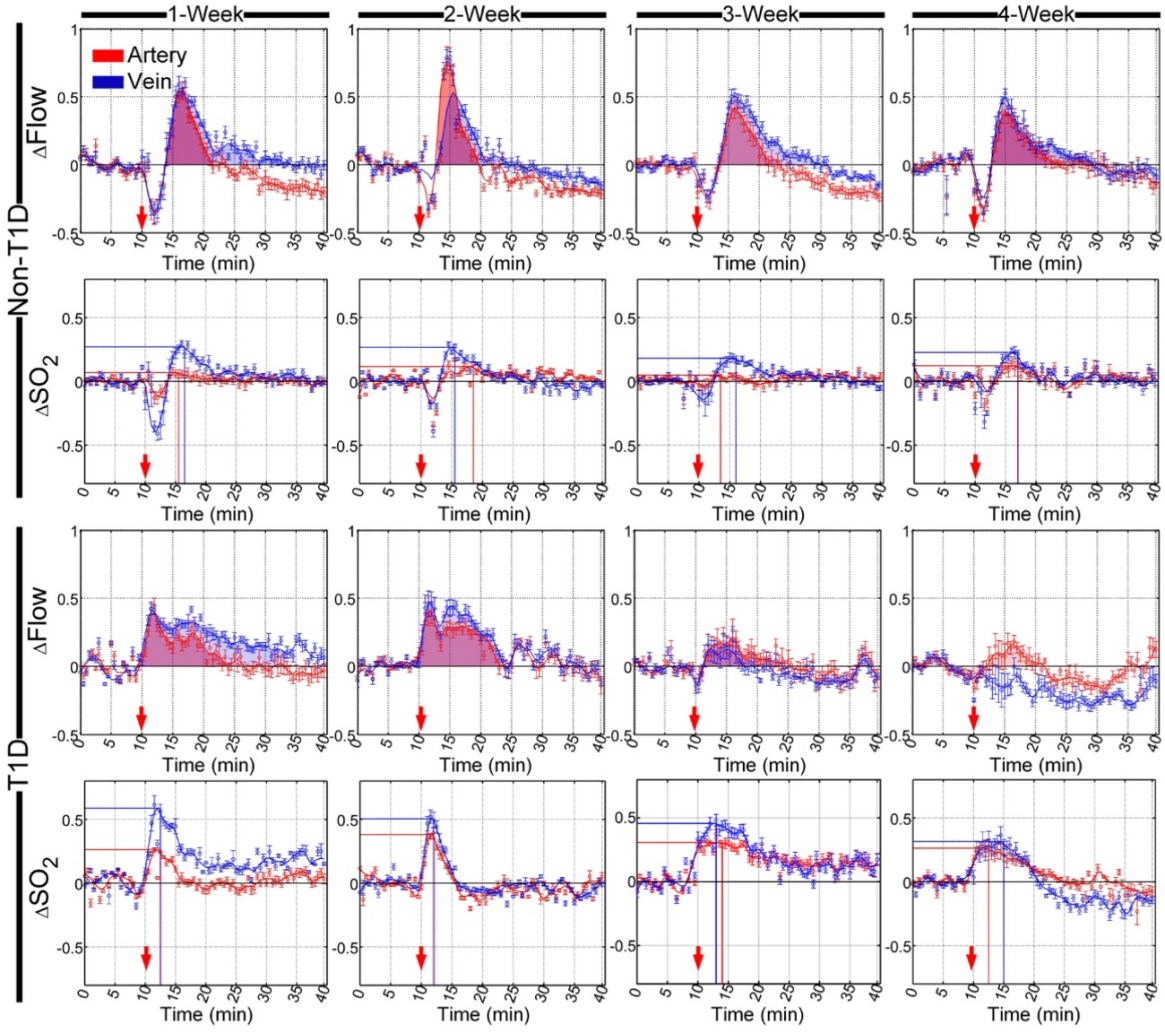
**Time-lapse data showing the relative changes in cerebral vascular blood flow and corresponding blood oxygen saturation that occurred in arteries (red) and veins (blue) after the injection of SNP in different stages of T1D.** The red arrows indicate the time of the injection. The shadowed areas indicate the areas under curves of relative changes in blood flow (red and blue represent arteries and veins, respectively). The lines perpendicular to the x- and y- axes represent the position of the maximum value of relative changes in blood oxygen saturation (red and blue lines indicate arteries and veins, respectively) (n=8, mean ± standard error).

SNP- and ACh-induced cerebral arteriovenous functional responses were dynamically monitored at different stages of diabetes development, and the cerebral arteriovenous △Flow and △SO_2_ recorded before and after the injection of SNP and ACh were quantitatively analyzed, as shown in **Figure 3** and** Figure S4**, respectively. In the non-T1D mice, cerebral microvascular △Flow quickly increased after an initial SNP-induced decrease, as shown in **Figure 3**, and the shapes of the time-lapse curves of △SO_2_ were slightly similar to those observed for △Flow. **Figure S4** shows that ACh caused △Flow and △SO_2_ to decrease. Additionally, the cerebral arteriovenous range of △SO_2_ was smaller than that of △Flow after SNP and ACh injection.

Besides, there were nearly no changes in the time-lapse curves of both △Flow and △SO_2_ among 1-, 2-, 3- and 4-week for the non-T1D mice.

In the T1D mice, the cerebral arteriovenous △Flow increased directly, and the initial decrease almost disappeared after the injection of SNP in the T1D mice at 1, 2, 3 and 4 weeks. In the 3- and 4-week T1D mice, the range of blood flow response induced by SNP became dramatically weak. Similarly, the diminished blood flow response after injection of ACh also was observed in the T1D mice (**Figure S4**). Compared to the non-T1D mice, during the development of T1D, the corresponding cerebral arteriovenous △SO_2_ also increased directly, and the initial decrease disappeared after the injection of SNP. Furthermore, in the T1D 1-, 2- 3- and 4-week mice, the increasing SNP-induced cerebral arteriovenous range of △SO_2_ was larger than that observed in the non-T1D mice (**Figure 3**). In addition, the ACh-induced decrease in △SO_2_ occurred only in veins, while the changes of △SO_2_ in artery almost disappeared in the T1D 1-, 2- 3- and 4-week mice (**Figure S4**). These data indicate that T1D can cause abnormal changes in cerebrovascular blood flow and blood oxygen responses at the early stage of T1D.

### Cutaneous arteriovenous functional response during the development of T1D

**Figure 4:**
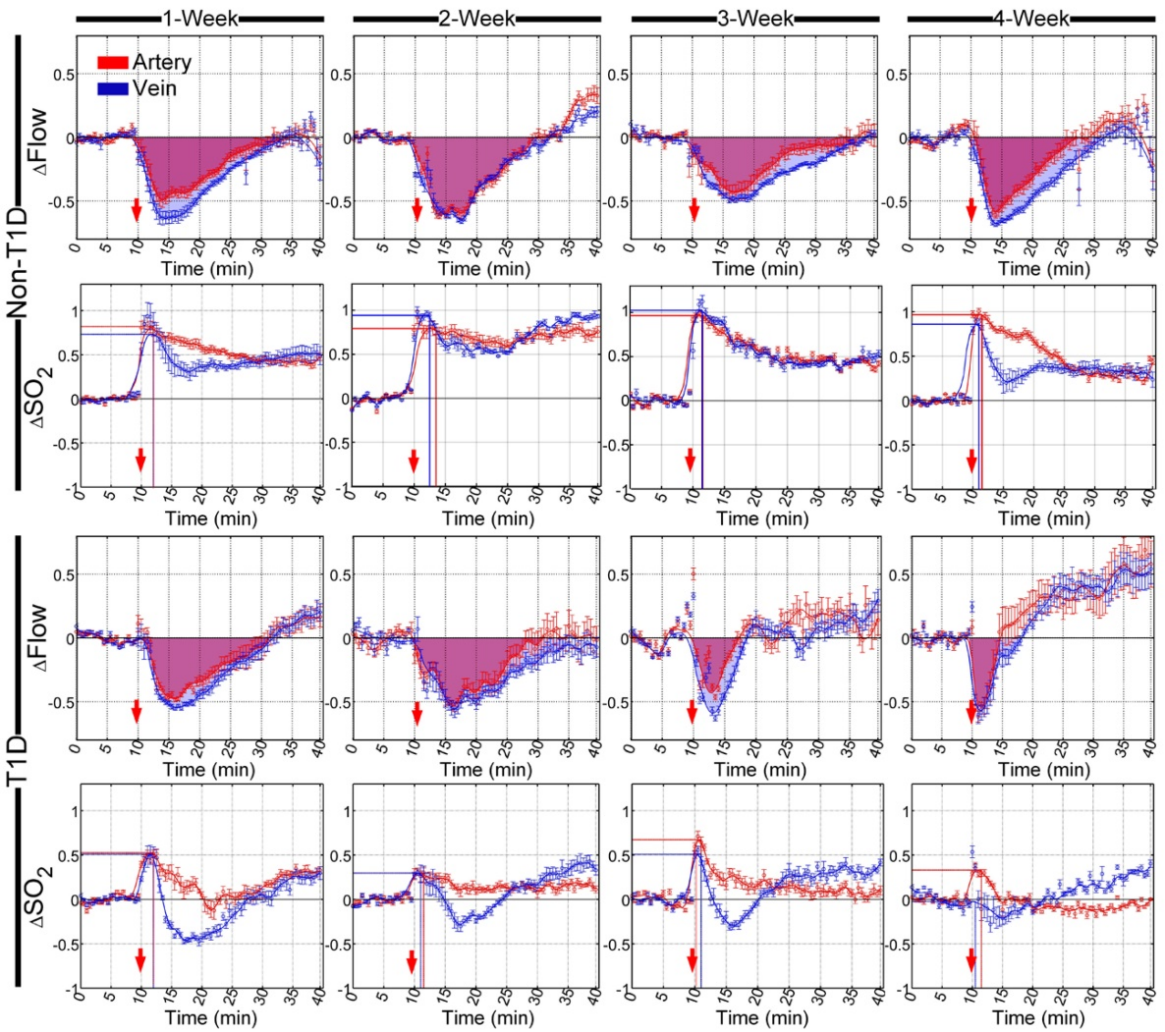
**Time-lapse of relative changes in cutaneous vascular blood flow and corresponding blood oxygen saturation in arteries (red) and veins (blue) after the injection of SNP at different stages of T1D.** The red arrows refer to the time of injection. The shadowed areas indicate the areas under curves of relative changes in blood flow (red and blue represent arteries and veins, respectively). The lines perpendicular to the x- and y- axes represent the position of the maximum value of relative changes in blood oxygen saturation (red and blue lines indicate arteries and veins, respectively) (n=8, mean ± standard error).

**Figure 5:**
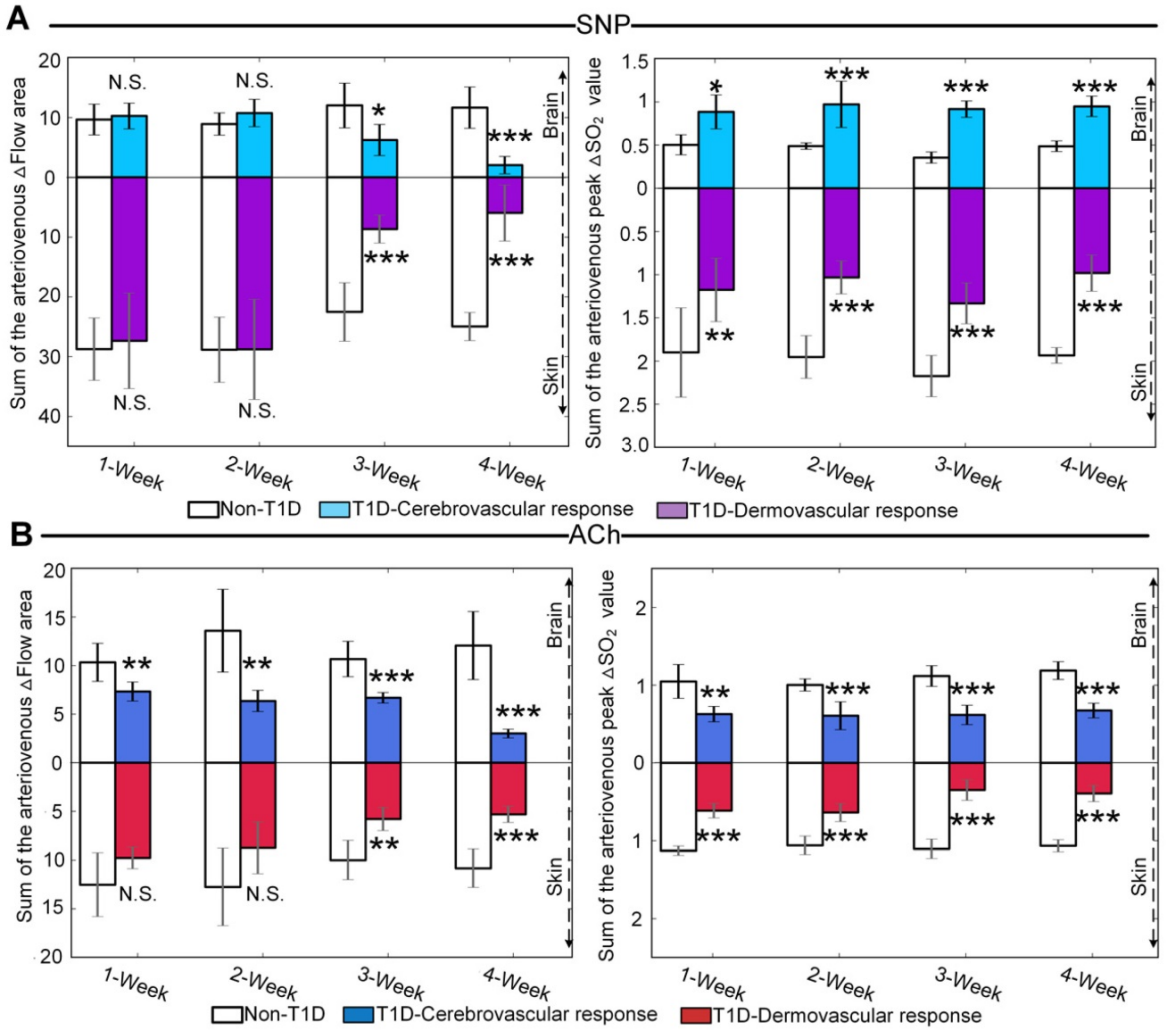
**Quantitative evaluation of cerebral and cutaneous microvascular function responses. (A)** Sum of the arteriovenous area of △Flow and sum of the arteriovenous peak values of △SO_2_ for SNP-induced microvascular response at different T1D stages (n=8).** (B)** Sum of the arteriovenous area of △Flow and sum of the arteriovenous peak values at decline of △SO_2_ for ACh-induced microvascular responses at different T1D stages (n=6). N.S., *, **, and *** indicate not significant, p < 0.05, p < 0.01, and p < 0.001, respectively, versus non-T1D (mean ± standard deviation).

Similarly, SNP- and ACh-induced cutaneous arteriovenous function responses were also dynamically monitored at different stages of diabetic development. Furthermore, quantitative analysis of cutaneous arteriovenous △Flow and the corresponding △SO_2_ before and after the injection of SNP and ACh are shown in **Figure 4** and** Figure S5**, respectively. In non-T1D, cutaneous arteriovenous △Flow decreased, while △SO_2_ increased after the injection of SNP, and the cutaneous microvascular time-lapse curves of △SO_2_ and △Flow were completely different. After the injection of ACh, the cutaneous arteriovenous △Flow and △SO_2_ both decreased, and the time-lapse curves of △Flow and △SO_2_ were similar (**Figure S5**). SNP injection induced a reduction in △Flow that recovered to the initial level after approximately 35 min, but the △SO_2_ did not recover until 40 min after the initial increase (**Figure 4**). Additionally, ACh caused a reduction in △Flow and △SO_2_, and both could recover to the initial level (**Figure S5**). In the non-T1D mice, there were almost no differences in the time-lapse curves of △Flow and the corresponding △SO_2_ among 1-, 2-, 3- and 4-week mice. **Figure 3** and **Figure 4** indicate that the SNP-induced arteriovenous blood flow and blood oxygen responses of skin microvessels were not consistent with those of cerebral microvessels. However, the trends in the blood flow and blood oxygen responses of skin microvessels induced by ACh were similar, as shown in **Figure 4S** and **Figure 5S**.

In T1D, after the injection of SNP and ACh, there were no obvious differences in cutaneous arteriovenous △Flow between T1D 1- and 2-week mice and non-T1D 1- and 2-week mice, respectively (**Figure 4** and** Figure S5**). However, in 3- and 4-week T1D mice, the recovery time of △Flow obviously advanced after stimulation with SNP and ACh. For the corresponding cutaneous arteriovenous △SO_2_, the SNP-induced increased amplitude of △SO_2_ observed in T1D was less than that observed in non-T1D from 1 to 4 weeks. After a rapid rise, the △SO_2_ of subcutaneous arteries gradually decreased and was nearly back to the initial level. However, in veins, the △SO_2_ gradually decreased and was lower than the initial level that occurred following the increase observed after the injection of SNP, then rose and became slightly higher than the initial level of the T1D mice from 1 to 3 weeks. In 4-week T1D mice, the rising peak observed on a time-lapse curve of subcutaneous venous △SO_2_ induced by SNP almost disappeared (**Figure 4**). Similarly, the ACh-induced decreasing amplitude of △SO_2_ in T1D was less than that observed in non-T1D from 1 to 4 weeks, especially for arteries, in which it almost disappeared in 3- and 4-week T1D mice (**Figure S5**). These results indicate that the abnormal changes in the cutaneous microvascular blood oxygen response caused by diabetes occur earlier than the changes in the blood flow response.

These findings indicate that T1D not only causes cutaneous microvascular dysfunction but also abnormal cerebral microvascular function responses in the early stages of T1D. In addition, the cutaneous microvascular trends observed in blood flow and the blood oxygen response to SNP were completely different, but they were similar to the cerebral microvascular responses. This indicates that there is a close correlation between cerebrovascular blood flow and blood oxygen, confirming previous reports 43.

### Quantitative comparison of cerebral and cutaneous microvascular functional response during the development of T1D

To quantitatively evaluate the differences between cerebral and cutaneous microvascular function responses, the sums of cerebral and cutaneous arteriovenous areas under/over the curves of △Flow, as indicated in red and blue shadows in **Figure 3**, **Figure 4** and **Figure S4**, **Figure S5**, were used for statistical analysis. Figure 5A shows that following SNP injection, there was no significant change in cerebral or cutaneous vascular responses between non-T1D and T1D mice after 1 week and 2 weeks, while the sums of cerebral and cutaneous arteriovenous areas of △Flow in the 3-week and 4-week T1D groups were significantly lower than those in the non-T1D groups. For ACh, **Figure 5B** shows that there was a significant difference in the cerebral blood flow response between non-T1D and T1D mice from 1 week to 4 weeks, whereas in the 3-week and 4-week groups, the sums of cutaneous arteriovenous areas of △Flow were significantly lower in the T1D groups than in the non-T1D groups. This indicates that T1D can cause systematic microcirculation disorders, such as cerebral and cutaneous microvascular abnormal responses in blood flow.

The sums of the arteriovenous peak values of △SO2 for cerebral and cutaneous microvascular responses are used for quantitative evaluation of vascular blood oxygen responses. For SNP and ACh, **Figure 5A-B** show that the sums of the arteriovenous peak values of cutaneous microvascular △SO2 for T1D groups significantly decreased compared to the non-T1D groups from 1 to 4 weeks. Following SNP induction, the sums of the arteriovenous peak values of △SO2 for cerebral blood oxygen responses in the T1D groups significantly increased (**Figure 5A**), while for ACh, these parameters in the T1D groups were significantly lower than those found in the non-T1D groups from 1 to 4 weeks (**Figure 5B**). This demonstrates that the cutaneous microvascular blood oxygen response becomes abnormal earlier than the blood flow response, indicating that the cutaneous vascular blood oxygen response to the reactivity test may serve as a good assessment indicator for evaluating cerebrovascular dysfunction in the early stage of diabetes. Additionally, the reduction in Ach- and SNP-induced blood flow responses implys that the functions of both the endothelium and smooth muscles are affected by T1D, consistent with a previous study 44.

### The expression levels of P-MLC and M3R in cerebral and cutaneous microvessels

**Figure 6:**
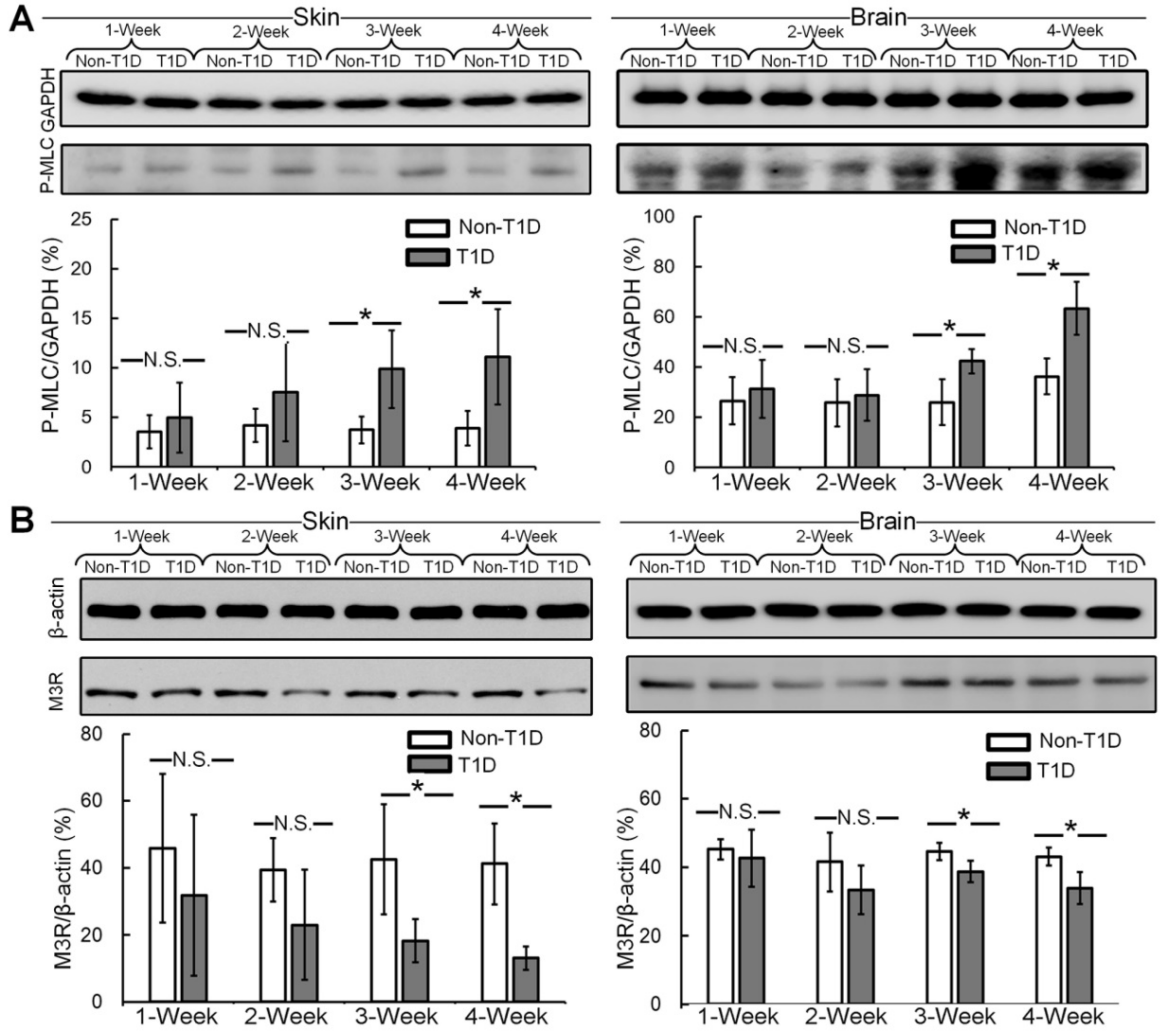
**Expression levels of P-MLC and M3R.** Representative immunoblots of P-MLC **(A)** and M3R** (B)** and the quantitation of the data obtained from western blot analysis. N.S. and * indicate not significant and p < 0.05, respectively, versus non-T1D (n=4, mean ± standard deviation).

Impairment of SNP-induced vasodilatation is correlated with enhanced vascular smooth muscle MLC phosphorylation in diabetes 45. Thus, the expression levels of P-MLC in cerebral and cutaneous vessels were analyzed by western blot. **Figure 6A** shows that in both the skin and brain, there were no significant differences in vascular smooth muscle MLC phosphorylation between non-T1D and T1D mice after 1 and 2 weeks. During the development of diabetes, in the skin and brain, vascular smooth muscle MLC phosphorylation was significantly higher at 3 and 4 weeks in the T1D groups than in the non-T1D groups. In addition, the endothelial M3R receptor subtype plays a major role in mediating cholinergic vasodilation 46. Therefore, we also analyzed the expression level of M3R in cerebral and cutaneous vessels by western blot. **Figure 6B** shows that the expression of M3R in the skin and brain was significantly lower after 3 and 4 weeks in the T1D groups than in the non-T1D groups.

The abnormal SNP- and ACh-induced blood flow responses appear to be associated with the upregulation of vascular MLC phosphorylation and the downregulation of endothelial M3R. Moreover, these results indicate that T1D can impair both cutaneous and cerebral vasodilatation.

### Physiological indexes of T1D mice

**Figure 7:**
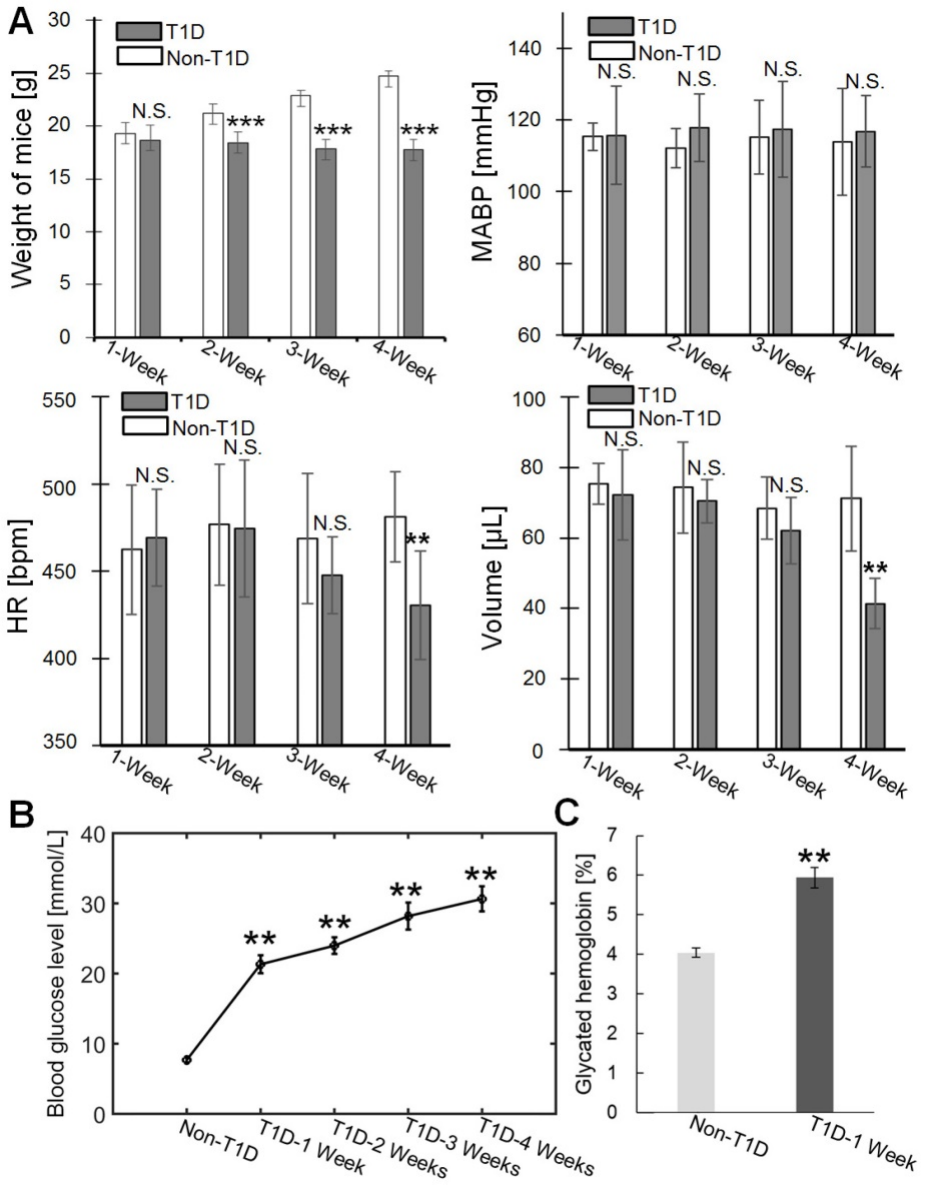
** Physiological indexes of mice in the various groups. (A)** Weight, mean arterial blood pressure (MABP), heart rate (HR) and blood volume at different stages of T1D (n=6). **(B)** Changes in blood glucose levels in the non-T1D group and the T1D groups (n=7). **(C)** Glycated hemoglobin levels in the non-T1D group and the 1-week T1D group. ** indicates p < 0.01 versus non-T1D. N.S., *, **, and *** indicate not significant, p < 0.05, p < 0.01, and p < 0.001, respectively, versus non-T1D (mean ± standard deviation).

The weight, blood pressure, heart rate and blood volume of the mice were measured at different stages of T1D, as shown in **Figure 7A**. The weight of T1D mice starts to decrease significantly from 2-week compared to non-T1D mice. There was no significant difference in MABP between the non-T1D mice and T1D mice from 1 to 4 weeks. The HR and blood volume of the T1D mice begin to significantly decrease at 4 weeks. In addition, the levels of blood glucose and glycated hemoglobin were also measured. **Figure 7B** shows that the vascular blood glucose levels were significantly higher in the T1D groups (1, 2, 3, and 4 weeks) than in the non-T1D group. Blood glucose and glycated hemoglobin levels showed a clear linear relationship 47. Therefore, only the glycated hemoglobin level for the 1-week T1D group was measured. **Figure 7C** shows that the glycated hemoglobin level in the 1-week T1D group was significantly higher than the level in the non-T1D group. This suggests that glycated hemoglobin levels were maintained at a high level during the progression of T1D. Vascular hemoglobin is the principal carrier of oxygen in the body 48. Thus, an increase in glycated hemoglobin levels could be related to the abnormal vascular blood oxygen response. Additionally, an increase in glycated hemoglobin can also quench nitric oxide 49, leading to dulled nitric oxide-dependent responses.

## Discussion

Diabetes-induced cerebrovascular disease can result in serious harm to humans, including an increased risk of stroke, cognitive decline and dementia 2-4. Therefore, special attention should be paid to affected patients 5-7. Currently, researchers usually use MRI to measure the cerebral blood flow level of diabetic patients in the resting state 7. However, these studies mainly focus on comparing differences between diabetic and normal states, and little attention has been paid to the dynamic changes that occur in cerebrovascular dysfunctions during the development of diabetes. A few studies have explored the impairment of cerebrovascular smooth muscle caused by T1D. All previously reported results are based on* ex vivo* cerebrovascular samples 6 or *in vitro* cerebrovascular smooth muscle cells 5.

The visualization of *in vivo* monitoring of cerebral and cutaneous microvascular reactivity with high spatial resolution is very important for a precise and objective analysis of vascular dysfunction. Optical imaging techniques represent important tools, but the turbid tissue limits the penetration of light. *In vivo* optical imaging usually depends on various windows 50, 51. Newly developed* in vivo* optical clearing techniques allow us to monitor subcutaneous and cortical microvascular blood flow and blood oxygen with high spatial resolution 27-29. In this study, HSI and LSCI were used for imaging vascular blood flow and blood oxygen through intact skin and skull. However, it's hardly to distinguish blood vessels due to the strong scattering of turbid tissue, especially in the skin. With the help of optical clearing skin and skull windows, the vascular blood flow and oxygen maps can be obtained clearly (**Figure S1**). To verify the effect of optical clearing methods on the vasculature, we monitored changes in cerebral and cutaneous vascular blood flow and oxygen saturation, as shown in **Figure S2** and **Figure S3**. We found that vascular blood flow and oxygen saturation remained almost constant during the observation period. Thus, the optical clearing methods did not affect vascular function. Compared to imaging windows based on surgery, *in vivo* optical clearing windows have some advantages, including a low cost, a simple operation and good safety 28, 37, 38. Specifically, in pathological conditions like diabetes, surgical sites do not easily heal and can even cause inflammatory stimulation or changes in the microcirculation. **Figure S1 A** shows that through the optical clearing skin window, the cutaneous capillary blood oxygen was narrowly detected by HSI due to the resolution of the detector, but the blood flow of capillaries was too low to be obtained by LSCI. Moreover, it was difficult to distinguish cortical capillaries, as shown in **Figure S1 B**. Thus, we analyzed non-capillary functional responses in this study.

Diabetes can cause various complications in many tissues and organs, such as the brain, eyes and skin 2, 52, 53. The skin is an accessible and generalized vascular bed. With the help of skin optical clearing and optical imaging techniques, Shi R.* et al.* monitored the recruitment and motility of immunocytes at different depths in skin inflammatory foci during the development of T1D 10 and found that abnormal changes occurred in the motility of immunocytes in deeper dermis at the early stage of T1D. In addition, Feng W. *et al*. also evaluated noradrenaline-induced skin vascular blood flow and oxygen responses during the development of T1D 9, and their results showed that diabetes induced abnormal changes in blood flow and oxygen. These results showed that T1D can lead to abnormal changes in the vascular and immune functions of skin, thus, the skin represents a very good research target. Some studies have indicated that cutaneous microvascular functions are damaged in the occurrence of some cardiovascular diseases, so the skin is also a potential representative vascular bed for the diagnosis of cardiovascular diseases 11-13. The diabetes-induced changes of vascular endothelial growth factor in skin and brain were consistent 18, 19. In addition, cutaneous vascular function has been used as a prognostic marker for evaluating the efficacy of drug effects on the microcirculation 15. Therefore, cutaneous vascular function has great potential as a research biomarker for predicting some diseases 14.

*In vivo* studies about the disorders of cutaneous vascular endothelium-dependent and endothelium-independent vasodilatation caused by diabetes are currently insufficient 54. In this work, we evaluated diabetes-induced damage to cutaneous microvascular vasodilatation *in vivo* and compared the results with those observed in disorders of cerebrovascular vasodilatation with the hope of identifying relationships for assessing cerebrovascular dysfunction. We found that compared to the occurrence of abnormal blood flow responses, SNP- and ACh-induced cerebral and cutaneous microvascular blood oxygen responses showed marked changes from T1D 1-week to T1D 4-week. This indicates that the cutaneous microvascular blood oxygen response to the reactivity test has the potential to be a good assessment strategy for evaluating cerebrovascular dysfunction in the early stage of diabetes. Furthermore, Gkogkolou P*. et al.* mentioned that changes in the skin could manifest before the onset of diabetes and might be related to complications in other internal organs, and this could be used as a basis for early diagnosis 55. In addition, a recent study reported that T1D rats showed coronary microvascular dysfunction as early as T1D 1-week 56, implying that microvascular dysfunction occurs relatively early in T1D animals. Therefore, we began to observe the vascular function response from 1-week TID, and long-term tracking studies need to be performed in the future.

It should be noted that SNP, as a common vascular stimulant, is used to test the vasodilatation of smooth muscle 57, and vascular smooth muscle plays an important role in the regulation of blood flow. Some previous studies have showed that the impairment of SNP-induced vasodilatation is correlated with the upregulation of vascular smooth muscle MLC phosphorylation in diabetes 45. Therefore, the expression levels of phosphorylated myosin light chain in the skin and brain were measured. The results were consistent with the changes observed in cerebral and cutaneous microvascular blood flow functional responses. Furthermore, ACh is a common dilator in most vascular beds and interacts with endothelial muscarinic receptors to enhance nitric oxide release, thus inducing vasodilation 15. The muscarinic acetylcholine receptors play an important role in modulating circulation, and the M3R receptor is important to mediating cholinergic vasodilation across five muscarinic receptor subtypes 46, 58. Here, the assessment of M3R levels indicated that T1D can lead to a decrease in M3R levels, and this could be one of the reasons for ACh-induced abnormal blood flow responses. Chen H. *et al.* also found that M3R was reduced in the seminal vesicles of diabetic rats. Generally, researchers pay more attention to the changes that occur in microvascular blood flow during SNP- and ACh-induced vasodilatation, but few studies have focused on the blood oxygen response. Philip E. James *et al.* showed that diabetes-induced glycosylation impaired the vasodilator function of red blood cells 59. Joshi, M. S. *et al.* and Teshima, Y. *et al.* showed that a hyperglycemia-induced increase in reactive oxygen species led to mitochondrial dysfunction in vascular smooth muscle and endothelial cells 56, 60. These will affect the vascular oxygen metabolism and damage the functional response of the vessels. Diabetes can cause a decrease in vascular endothelial nitric oxide synthase, which can alter the oxygen consumption. Therefore, nitric oxide plays an important role in matching blood flow to tissue metabolism 54, 61. In addition, T1D caused a decrease in HR and blood volume in 4-week T1D mice, consistent with previous studies 62. It may also further aggravate the abnormal changes that occur in the vascular response of 4-week T1D mice. There was no significant difference in weight between 1-week T1D mice and 1-week non-T1D mice, while the growth of the mice was affected beginning at 2 weeks T1D. Our results show that the vascular blood oxygen and blood flow responses of non-T1D mice did not substantially change with age. Therefore, these data indicate that the changes in body weight observed in T1D mice may not directly affect vascular functional responses.

Vascular hemoglobin, as the principal carrier of oxygen, is related to the vascular blood oxygen response. In persistent hyperglycemia, glycated hemoglobin will also be maintained at a high level, and this can affect the vascular blood oxygen response. Our results further demonstrate that there are dramatic changes in cerebral and cutaneous vascular blood oxygen responses even in the early stages of T1D, and these may also be associated with the increase in glycated hemoglobin caused by diabetes. A recent study showed that the inner retinal oxygen metabolic rate increased in early diabetes, while anatomical retinal vascular complications were not detected, and changes in the oxygen metabolic rate occurred before changes in the blood flow rate 63. This finding is consistent with the changes in cutaneous oxygen metabolism observed in this study. Additionally, our results show that there are some differences in the extent of changes in arteriovenous blood oxygen caused by T1D between cerebral and cutaneous vessels, potentially because the brain needs enough oxygen to maintain its normal function 64. Some diseases, such as diabetes, can impair the important link between cerebral blood flow nutrient supply and metabolic demands, and this may affect cerebral autoregulation 65. However, the hypoxia in skin tissue caused by diabetes may aggravate the abnormal vascular blood oxygen response 66. Our results show that there was an overall difference in the SNP-induced vascular response between cerebral and cutaneous microcirculation in non-T1D mice. However, Ach-induced cerebral blood flow and oxygen responses were similar to those observed in cutaneous vessels in the non-T1D mice. Chang C. L.* et al.* also showed that there is a difference between the cerebral and peripheral vessels in the blood flow responses to SNP 67. In addition, Gustafss U. *et al.* showed that the changes in blood flow induced by ACh occurred parallel to changes in blood oxygen demand, but it was non-parallel for the SNP induction in the skeletal muscle 68. This finding is similar to our results regarding SNP- and ACh-induced cutaneous vascular responses.

In this study, the 2-week T1D mice were managed with insulin for one week. Then, we measured physiological indexes and monitored SNP- and ACh-induced changes in cerebral and cutaneous vascular blood flow and blood oxygen responses, as shown in **Figure S6**. Insulin treatment effectively prevented 2-week T1D mice from losing weight as the T1D progressed, and there were no changes in systemic hemodynamics (**Figure S6 A**). **Figure S6 B** shows that after insulin treatment for one week, the SNP- and ACh-induced cerebral and cutaneous vascular responses of 2-week T1D mice did not change in a manner similar to that observed in the 3-week T1D groups, indicating that insulin treatment can effectively intervene in the development of diabetes. This is consistent with previous reports 69 that indicated that blood sugar control with insulin plays an important role in restraining the vascular dysfunction caused by T1D.

## Conclusion

In this work, *in vivo* monitoring of SNP- and ACh-induced cerebral and cutaneous microvascular responses (including blood flow and blood oxygen saturation) during the development of T1D was performed with the assistance of *in vivo* skull and skin optical clearing techniques. When we compared the differences between cerebral and cutaneous vascular dysfunction, we found that in the early stage of diabetes (1 week), abnormal changes occurred in cerebral vascular blood flow and blood oxygen responses as well as cutaneous vascular blood oxygen. With the development of T1D, the cutaneous vascular blood flow response also became abnormal. These results suggest that the cutaneous vascular blood oxygen response was more sensitive than blood flow to the reactivity test, and therefore has the potential to serve as a good assessment indicator for revealing cerebrovascular dysfunction in the early stage of diabetes. Furthermore, we explored the reasons for changes in vascular function responses and found that it was associated with changes in vascular P-MLC and M3R levels. Additionally, the abnormal blood oxygen responses could be related to the increase in glycated hemoglobin levels. Our results indicate that cutaneous microvascular function can be a potential research biomarker for early warning in diabetes-induced cerebrovascular dysfunction, and therefore has great clinical value for the diagnosis and medical intervention in the early stages of diabetes.

## Supplementary Material

Supplementary figures and tables.

## Abbreviations

ACh: acetyl choline
CCD: charge coupled device
△Flow: relative changes in blood flow
GAPDH: glyceraldehyde 3 phosphate dehydrogenase
HSI: hyperspectral imaging
HR: heart rate
LSCI: laser speckle contrast imaging
LCTF: liquid crystal tunable filter
MRI: magnetic resonance imaging
MLC: myosin light chain
M3R: muscarinic acetylcholine receptor M3
MABP: mean arterial blood pressure
P-MLC: phosphorylated myosin light chain
SNP: sodium nitroprusside
△SO_2_: relative changes in blood oxygen saturation
T1D: type 1 diabetes
