# Supplementary material for: Comparison of cerebral and cutaneous microvascular dysfunction with the development of type 1 diabetes
Source: Theranostics. 2019 Aug 12;9(20):5854–68. doi: 10.7150/thno.33738 (PMC6735377; doi:10.7150/thno.33738)
Supplement: Supplementary file 1 — Supplementary figures and tables. [file thnov09p5854s1.pdf]

SUPPLEMENTARY MATERIALS

Comparison of cerebral and cutaneous microvascular dysfunction with the development of type 1 diabetes

Wei Feng [1, 2], Shaojun Liu [1, 2], Chao Zhang [1, 2], Qing Xia[1, 2], Tingting Yu [1, 2] and Dan Zhu [1, 2]✉

1. Britton Chance Center for Biomedical Photonics, Wuhan National Laboratory for Optoelectronics-Huazhong University of Science and Technology, Wuhan, Hubei 430074, China
2. MoE Key Laboratory for Biomedical Photonics, Huazhong University of Science and Technology, Wuhan, Hubei 430074, China

✉ Corresponding author: Dan Zhu, Britton Chance Center for Biomedical Photonics, Wuhan National Laboratory for Optoelectronics, Huazhong University of Science and Technology, Wuhan 430074, China. Tel: 86-027-87792033; E-mail: dawnzh@mail.hust.edu.cn.

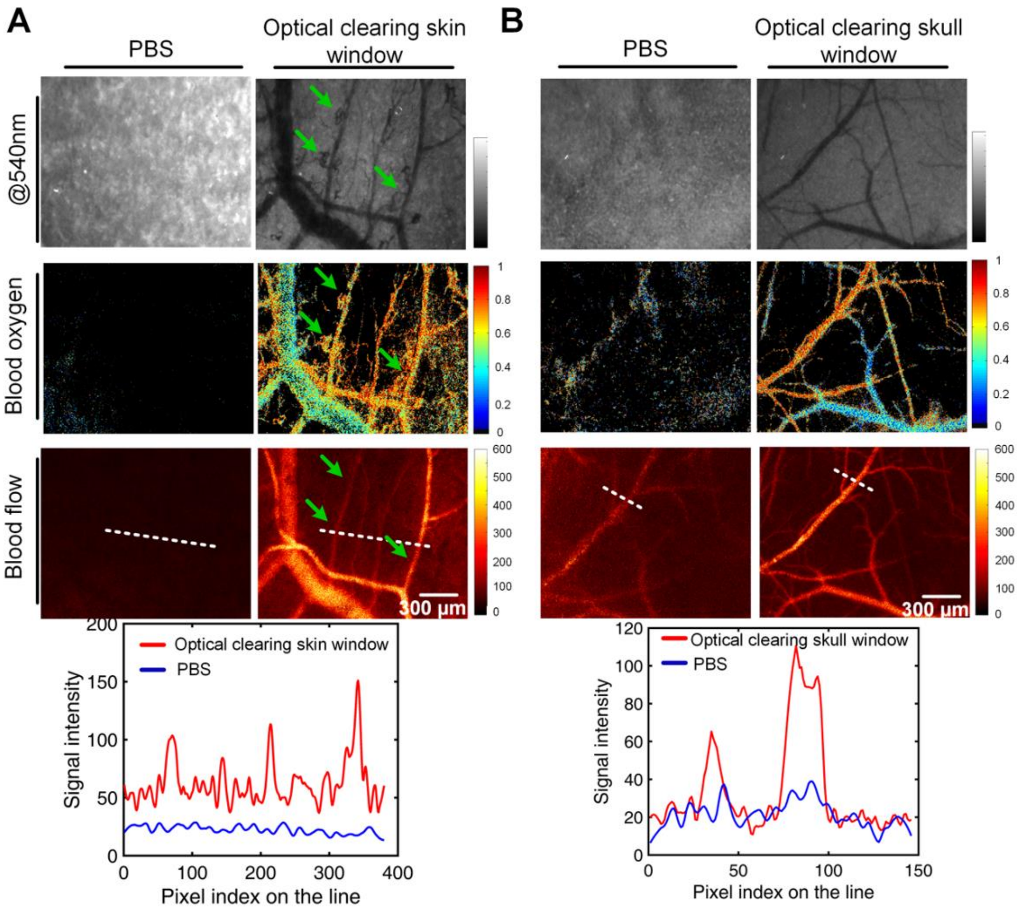

Figure S1. Imaging the cutaneous and cortical microvessels with the help of optical clearing skin window (A) and optical clearing skull window (B). First row: the images at 540 nm; Second row: blood oxygen maps obtained by HSI; Third row: blood flow maps obtained by LSCI; Fourth row: the signal intensity along the white dashed lines in the blood flow images. First column: cutaneous imaging through skin treated with PBS; Second column: imaging cutaneous vessels through optical clearing skin window; Third column: cortical imaging through skull treated with PBS; Fourth column: imaging cortical vessels through optical clearing skull window. (The green arrows in **A** indicate the capillaries of the skin)

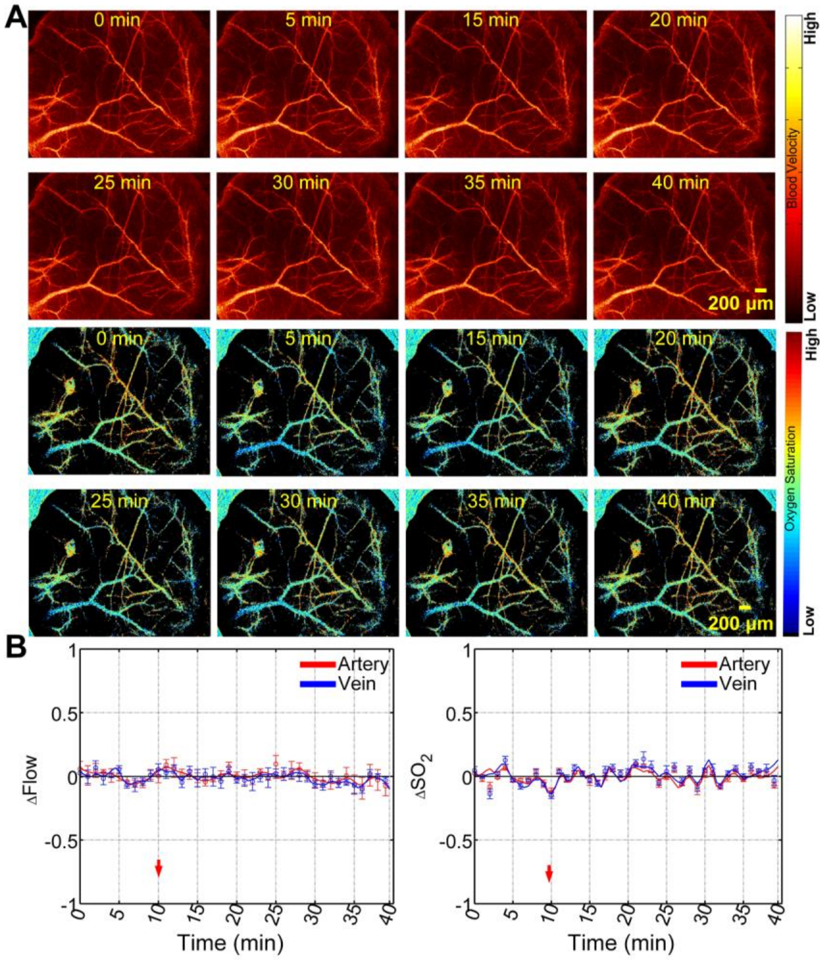

Figure S2. Monitoring changes of cerebral vascular blood flow and corresponding blood oxygen saturation after injection of normal saline. (A) Typical blood flow and blood oxygen saturation maps of cerebral vessels through optical clearing skull window. **(B)** The relative changes of cerebral vascular blood flow and corresponding blood oxygen saturation in the artery and vein after injection of 125 μL normal saline (n=5, mean ± standard error).

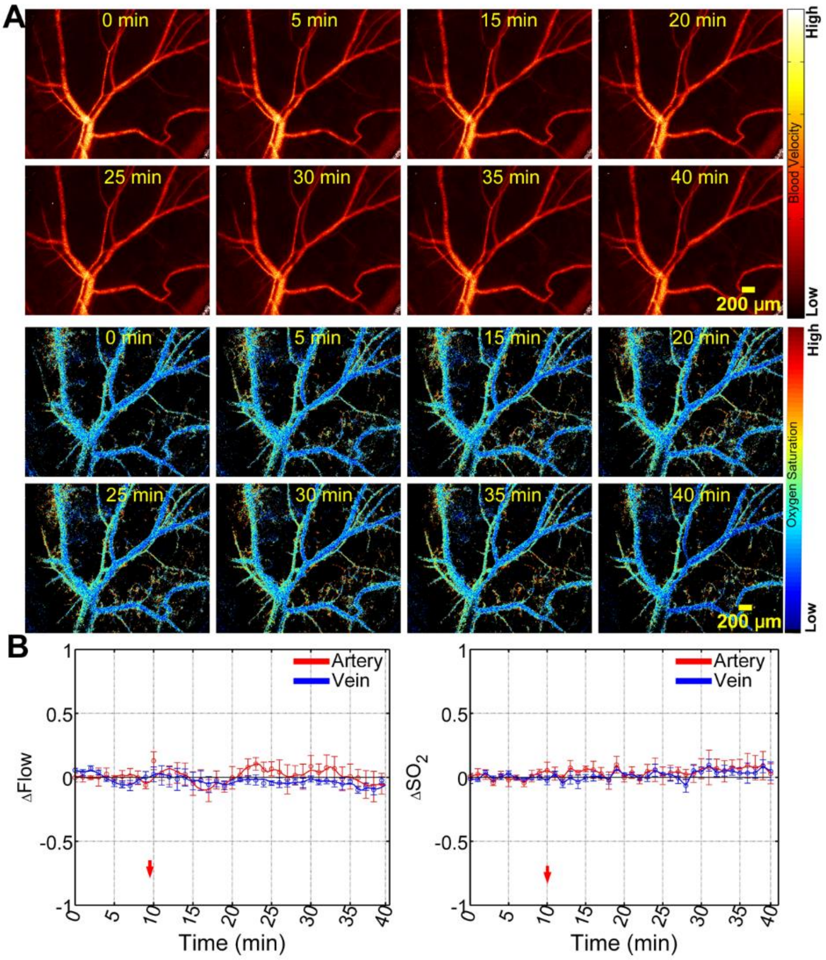

Figure S3. Monitoring changes of cutaneous vascular blood flow and corresponding blood oxygen saturation after injection of normal saline. (A) Typical blood flow and blood oxygen saturation maps of cutaneous vessels through optical clearing skin window. (B) The relative changes of cutaneous vascular blood flow and corresponding blood oxygen saturation in the artery and vein after injection of 125 μL normal saline (n=5, mean ± standard error).

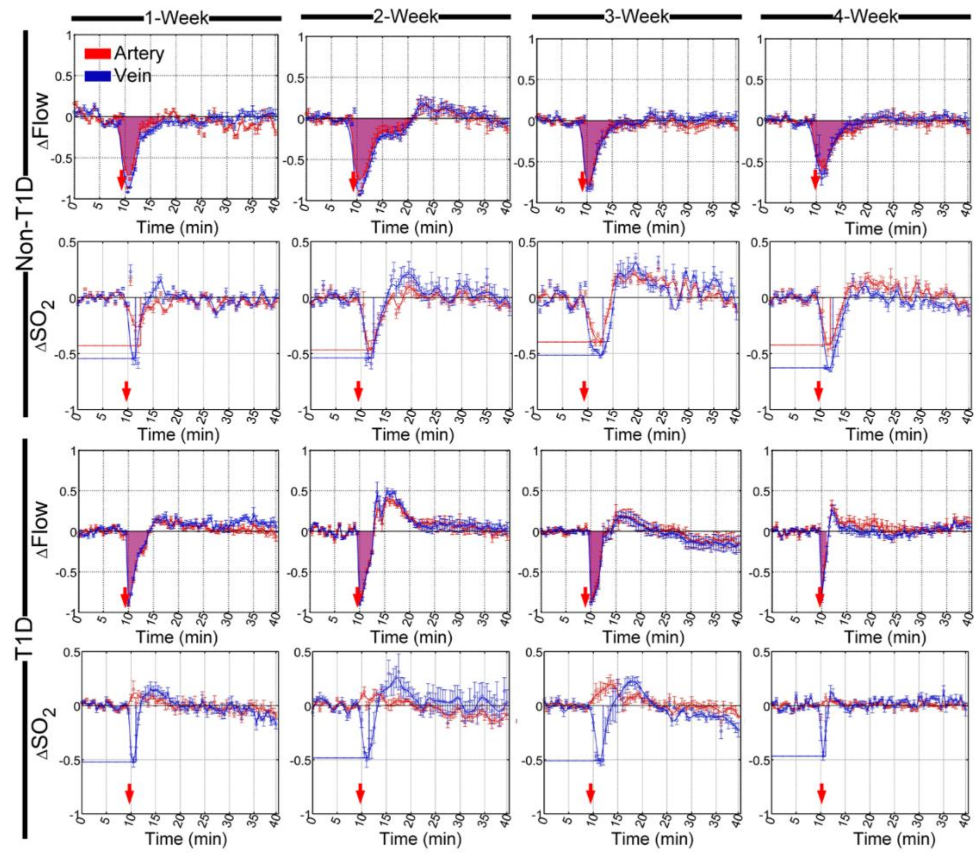

Figure S4. Time-lapse data showing the relative changes in cerebral vascular blood flow and corresponding blood oxygen saturation that occurred in arteries (red) and veins (blue) after the injection of ACh in different stages of T1D. The red arrows refer to the time of injection. The shadowed areas indicate the areas under curves of relative changes in blood flow (red and blue represent arteries and veins, respectively). The lines perpendicular to the x- and y- axes represent the position of the maximum value of relative changes in blood oxygen saturation (red and blue lines indicate arteries and veins, respectively) (n=6, mean ± standard error).

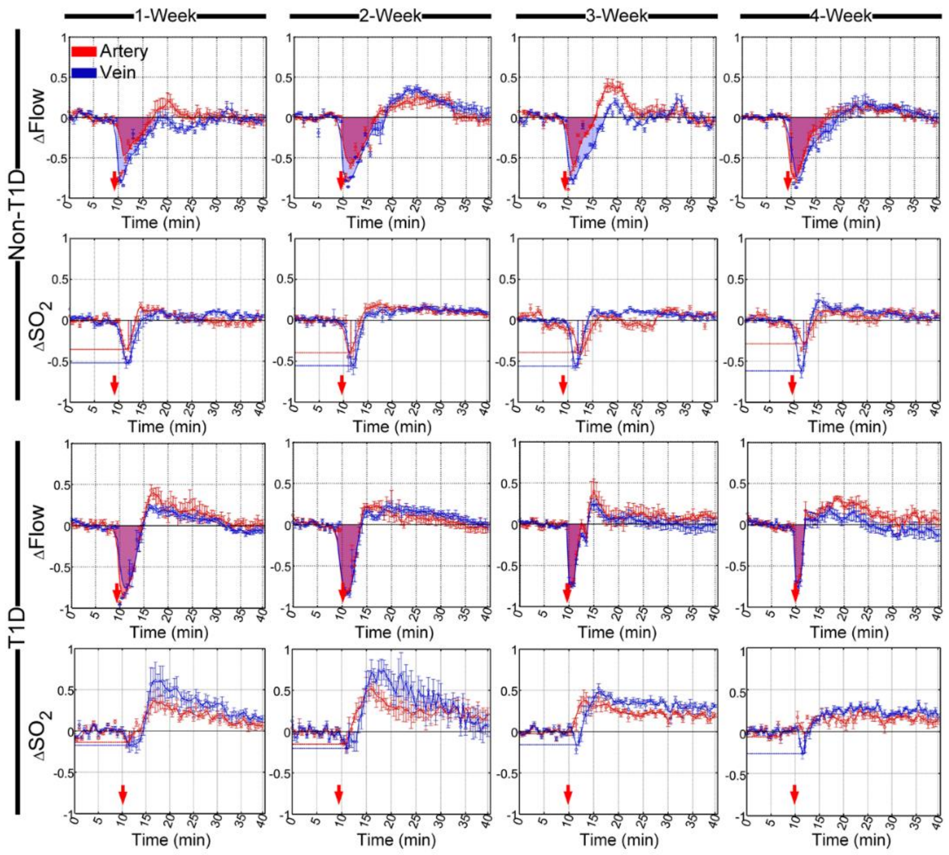

Figure S5. Time-lapse of relative changes in cutaneous vascular blood flow and corresponding blood oxygen saturation in arteries (red) and veins (blue) after the injection of SNP at different stages of T1D. The red arrows refer to the time of injection. The shadowed areas indicate the areas under curves of relative changes in blood flow (red and blue represent arteries and veins, respectively). The lines perpendicular to the x- and y- axes represent the position of the maximum value of relative changes in blood oxygen saturation (red and blue lines indicate arteries and veins, respectively) (n=6, mean ± standard error).

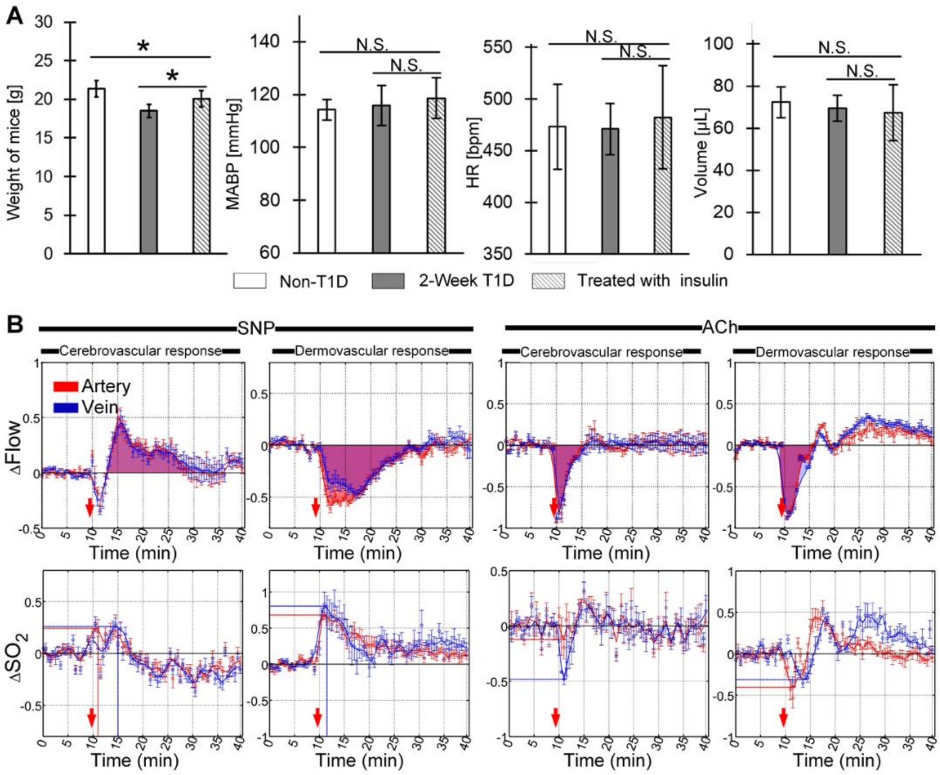

Figure S6. Physiological indexes and arteriovenous vascular functional responses of the 2-week T1D mice managed with insulin for one week. (A) The weight, mean arterial blood pressure, heart rate and blood volume. **(B)** The time-lapse relative changes of cerebral and cutaneous vascular blood flow and corresponding blood oxygen saturation in the artery (red) and vein (blue) after injection of SNP and ACh. N.S. and * indicate not significant and $p < 0.05$, respectively, versus non-T1D (n=6, mean ± standard deviation).
